# A MRI study of the lesser trochanteric version and its relationship to proximal femoral osseous anatomy

**DOI:** 10.1093/jhps/hnv067

**Published:** 2015-11-09

**Authors:** Ricardo Gonçalves Schröder, Manoj Reddy, Munif Ahamad Hatem, Juan Gómez-Hoyos, Leon Toye, Anthony Khoury, Hal David Martin

**Affiliations:** 1. Hip Preservation Center at Baylor Scott&White Health Dallas, TX, USA; 2. Texas A&M Health Science Center of College of Medicine, Dallas campus, TX, USA; 3. Universidade Federal do Parana, Curitiba, PR, Brazil; 4. Department of Radiology, Baylor Scott&White Health Dallas, TX, USA; 5. Bioengineering Department, University of Texas at Arlington. Arlington, TX, USA

## Abstract

The purpose of this study is to quantify the lesser trochanteric version and determine the angle and the relationship between lesser trochanter and femoral neck version. Investigate the influence of the lesser trochanter version in the width of ischiofemoral space. Two hundred and fifty asymptomatic hips were evaluated with axial magnetic resonance image. The lesser trochanter version was calculated. The difference between the femoral neck version and the lesser trochanter version formed the angle between each structure. The width of ischiofemoral space was measured and its relationship with the lesser trochanter version was determined. The mean lesser trochanter version was −24° ± 11.5° (range, − 54° to + 17°) with a coefficient variation of 47.45%. The mean femoral neck version measured 14.0° ± 10.8° (range, −16° to 50°), with a coefficient variation of 81.32%. The lesser trochanter/femora neck angle was 38.4° ± 9.6° (range, 8° to 67°), coefficient variation of 30%, with a moderate correlation between the structures (*r* = 0.63, *P* < 0.01). The mean ischiofemoral space was 22.9.0 ± 7.0 mm (range, 10.3 to 55 mm), and a weak correlation was found between ischiofemoral space and lesser trochanteric version (*r* = −0.16, *P* < 0.05). The lesser trochanteric version showed a high variation with a moderate relationship with the femoral neck version. The lesser trochanteric version does not influence the width of the ischiofemoral space.

## INTRODUCTION

The importance of the lesser trochanter (LT) and its clinical implications have been increasingly explored in the literature [1–7]. Impingement of the LT against the ischium is recognized as a cause of posterior hip pain and the retroversion of the LT has been associated to development of snapping hip [3, 6, 8]. LT anatomy has also considered in reconstruction surgeries, since the LT is utilized as a guide for the placement of the femoral component in an appropriate anteversion [1, 9].

**Fig. 1.**
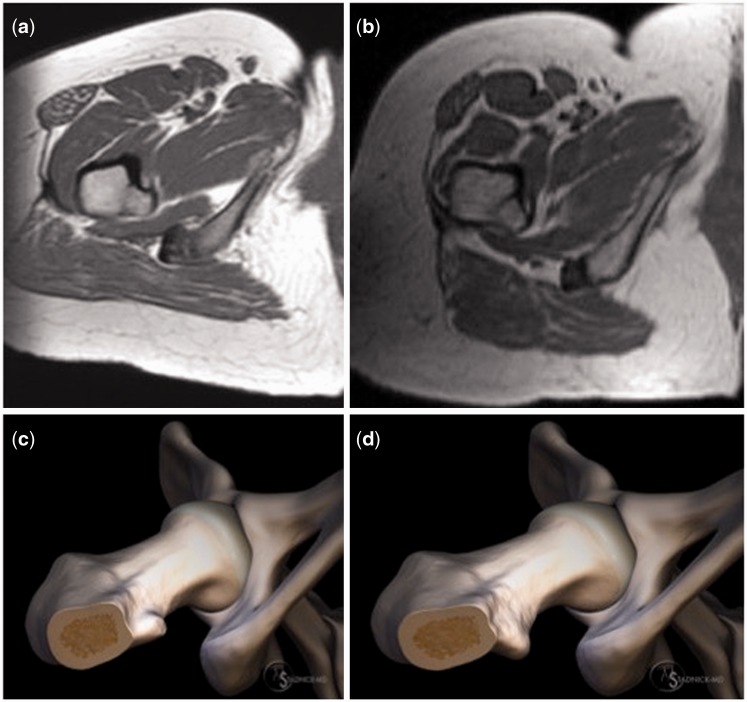
Axial T1-weighted MR images and graphic illustrations demonstrating two different lesser trochanteric versions. (a) and (c) demonstrate a typical lesser trochanteric version, whereas (b) and (d) demonstrate a retroverted LT.

 The ischiofemoral space (IFS) was defined on magnetic resonance axial images as the smallest distance between the ischial tuberosity and the LT. An IFS narrower than 17 mm would be a diagnostic feature for ischiofemoral impingement [10]. Factors in the measurement of IFS are leg positioning and alterations of proximal femoral and pelvic anatomy, which may contribute to decreased IFS and effect the development of IFI [3, 11–14]. The proximal femur has also involved in the development of snapping hip and outcomes of psoas lengthening in hip arthroscopy [6, 15]. Lesser trochanterplasty and release of the iliopsoas tendon at the LT has been shown as an effective treatment of IFI and iliopsoas snapping [5, 8, 16, 17]. Although lesser trochanterplasty in confirmed cases of IFI has had good outcomes, the influence of anatomical variations of the LT upon the IFS and the relationship with the proximal femoral anatomy is not well known (Fig. 1).

The purposes of this investigation were to: (i) Investigate the LT version (LTV) related to the posterior femoral condyles in asymptomatic magnetic resonance images (MRIs). (ii) Quantify the angle and relationship between the axis of the LT and the femoral neck (FN) (LTFN angle). (iii) Investigate the influence of LTV on the IFS.

## MATERIAL AND METHODS

### Subjects and MR

A retrospective analysis was performed in 320 hip MRIs in patients who underwent MRIs for contra-lateral hip pain. All MRIs were performed on 1.5 Tesla MRI scanners (Intera Nova and Achieva, Philips Medical Systems, Netherland) utilizing a phased array coils. In all cases, a straight (non-obliqued) axial MR sequence was performed which included the bilateral hips within the field-of-view. In addition, axial cuts at the distal femur were obtained with both lower extremities taped in internal rotation [18–20]. Exclusion criteria included: MRI studies without axial cuts through the entire LT or the absence of MRI axial cuts through the femoral condyles; signs of trauma and previous surgery on studied side.

### Measurements

Measurements were obtained on straight (non-obliqued) axial images, utilizing the software Virtual Radiology Enterprise Connect PACS (Philips Healthcare Informatics, Inc.).

#### LT version

**Fig. 2.**
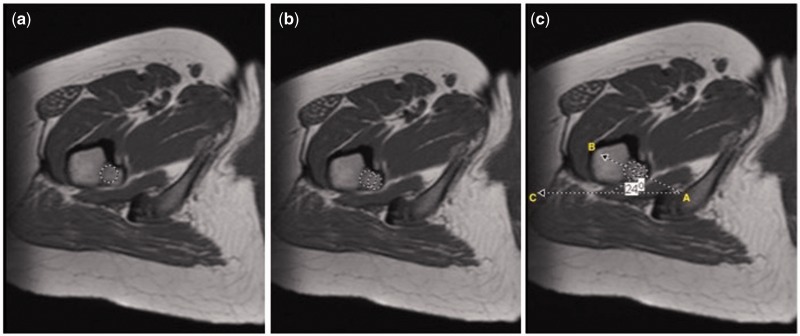
Axial T1-weighted MR images demonstrating a two-centroid method to measure lesser trochanteric version: An image demonstrating the largest width of the LT is selected. (a) The first centroid is placed at the midline lesser trochanteric base; (b) The second centroid is placed at the border of the lesser trochanteric tip; (c) Axis of LT represented by the line AB; the horizontal line is represented by the line AC. The angle CAB forms the LT axis.

**Fig. 3.**
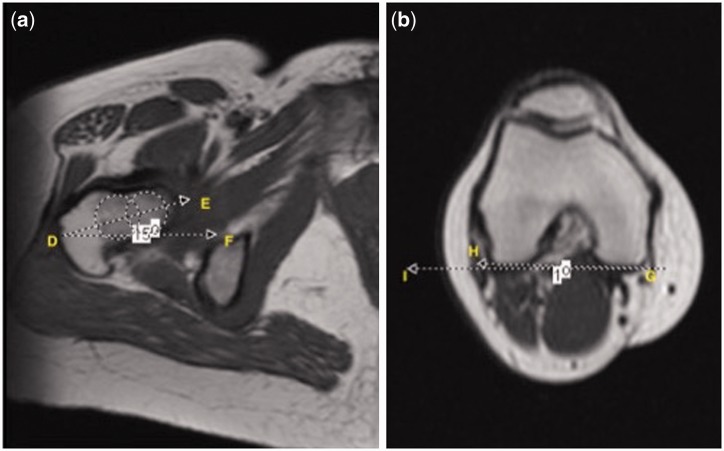
Axial T1-weighted MR image shows the representation of the: (a) Angle DEF representing the Femoral Neck axis. Two centroids were positioned through the femoral neck shaft. FNa was formed by the angle of the line passing through the middle of the both centroids relative to the horizontal line; (b) posterior condylar axis of the knee as the angle IGH.

The LT axis and posterior surface of the femoral condyles were utilized to determine the LTV. On the MRI slice showing the largest width of the LT [1], two centroids were positioned on the LT: the first at the midline of the base (Fig. 2a) and the second at the border of the tip (Fig. 2b). A line passing through the center of both centroids corresponded to the LT axis. LTV was defined as the angle between the LT axis and a line passing at the posterior surface of the femoral condyles (Fig. 3a) [18].

#### LTFN angle

**Fig. 4.**
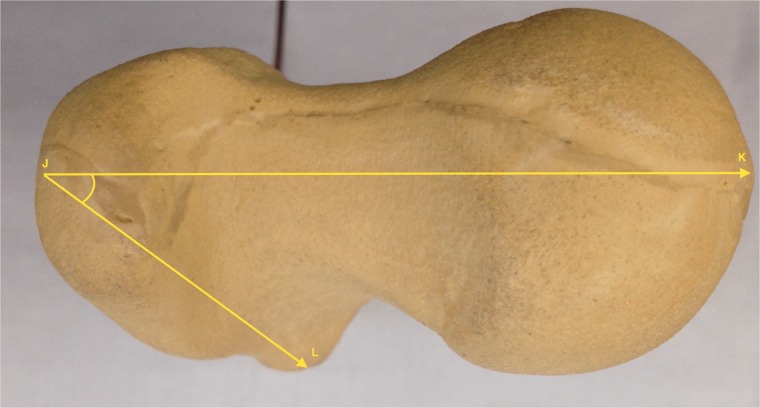
Gross anatomic axial view of the proximal femoral neck and head. The relationship between FN axis and LT axis is represented by the angle KJL.

**Fig. 5.**
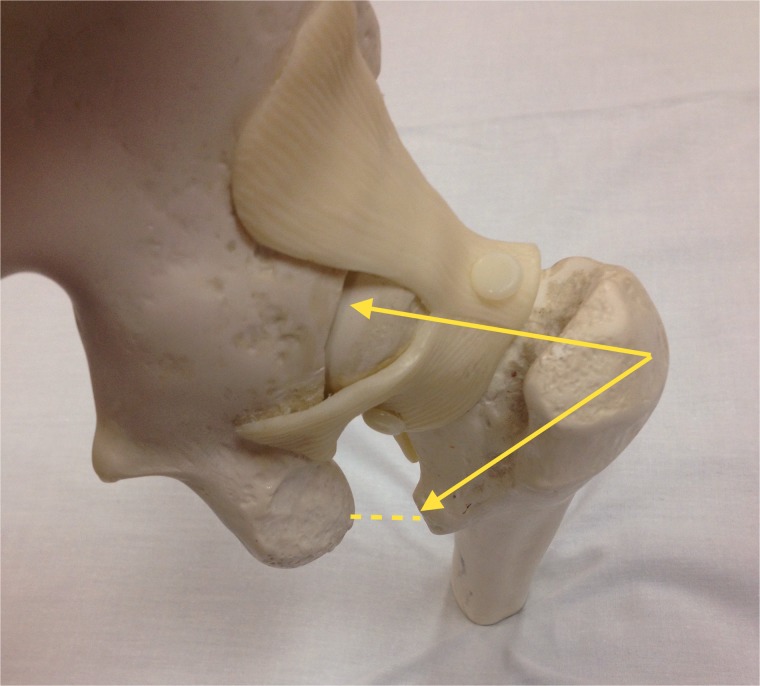
Gross anatomic view of the hip. The relationship between the orientation of the LT, femoral neck version and the IFS.

The FN axis was assessed to obtain the LTFN angle. The MRI axial slice taken just below the femoral head was utilized to measure the FN version (FNV) [21]. Two centroids were positioned on the neck of the femur and a line passing through the center of both centroids corresponded to the FN axis. FNV was defined as the angle between the FN axis and a line passing at the posterior surface of the femoral condyles [18]. Finally, the FNLT angle was calculated by the formula: FNLT angle = FNV–LTV (Figs 4 and 5).

#### Influence of the LTV on the IFS

**Fig. 6.**
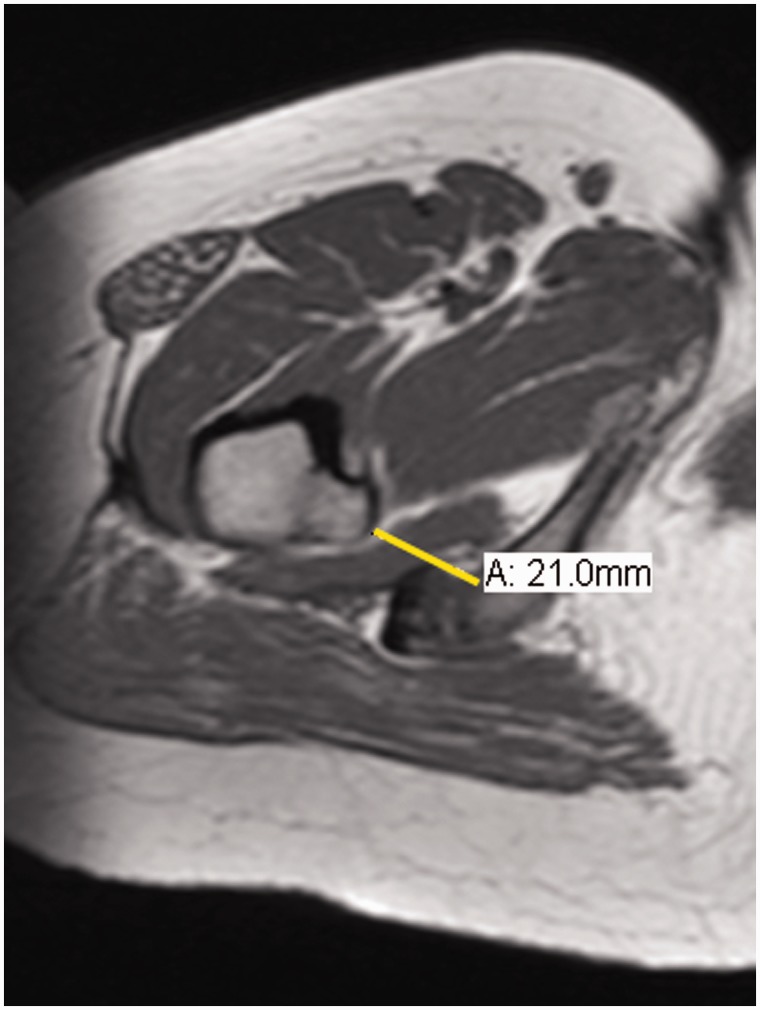
Axial T1-weighted MR image shows typical IFS measurement (line A).

The IFS measurements were conducted according to the method previously described by Torriani *et al.* [10] (Fig. 6). According to the author the IFS was defined as the smallest distance between the lateral cortex of the ischial tuberosity and medial cortex of the LT expressed in millimeters.

For all methods two researchers (R.S. and M.R.) performed the measurements separately. One computer was used to perform all measurements. Both examiners were blind to all clinical-patient data. Each researcher performed two measurements of the same pre-selected image, with 2 weeks between each measurement.

### Statistical analysis

Intra-class coefficient correlations (ICCs) were calculated to determine intra- and inter-observer agreements (SPSS IBM V.14). All data are presented as mean ± standard deviation (SD), and range. Independent *t*-tests were used to determine differences. ICCs, with a 95% confidence interval, were calculated based on 30 repeated measurements to determine intra- and inter-examiner agreement. Level of significance was set to *P* ≤ 0.05. The ICCs for all measurements by examiner 1 ranged from 0.896 to 0.923 and examiner 2 ranged from 0.788 to 0.952. The ICCs for both examiners together ranged from 0.826 to 0.906.

## RESULTS

Of the 320 MRI analysed, 70 subjects were excluded. Two hundred and fifty (250) MRIs were included in this study. The subjects were 164 females (65.4%) and 86 males (34%), with an average age of 39.5 years.

The mean LTV was −24° ± 11.5° (range, −54° to +17°) with a coefficient variation of 47.45% and the mean FNV was 14.0° ± 10.8° (range, −16° to 50°), coefficient variation of 81.32%. The average for the LTFN angle was 38.4° ± 9.6° (range 8° to 67°), coefficient variation of 30% with a moderate correlation between the LTV and FNV (*r* = 0.63, *P* < 0.01). The mean IFS was 22.9.0 ± 7.0 mm (range, 10.3 to 55 mm), and a weak correlation was found between IFS and LTV (*r* = −0.16, *P* < 0.05). No statistically significant difference was found between male and female in the assessed parameters for LTV and its relationships (Table I).

**Table I.** Demographic data and results of measurements

	Male (*n* = 86)		Female (*n* = 164)	
Variable	Mean ± SD	Range	Mean ± SD	Range
**Age (years)**	41.6 ± 14.9	15 to 73	38.4 ± 14.7	14 to 69
**LTV (degrees)**	−23.6 ± 12.0	−49 to 9	−24.5 ± 11.3	−54 to 17
**FNV (degrees)**	14.0 ± 10.3	−1 to 43	14.2 ± 11.0	−16 to 50
**FNLTa (degrees)**	37.65 ± 10.4	8 to 60	38.8 ± 9.2	13 to 67
IFS* (mm)	27.5 ± 7.4	15 to 56	20.5 ± 5.4	10 to 35

LTV, LT version; FNV, femoral neck version; FNVLTVa, femoral neck version lesser trochanter version angle; IFS, ischiofemoral space; *P < 0.001.

## DISCUSSION

The LT presented a posterior orientation related to the distal femur in most of the 250 hips with an average LTV of −24°. The variation of LTV was high (47.45%), which included hips with the LT anteriorly directed (anteverted) in relation to the distal femur. There was a positive relationship (30%) between LTV and FNV with the angle formed between each structure averaging 38.4° ± 9.6° (range, 8° to 67°). The moderate variation of LTFN angle could be explained due the high variability (81.32%) of FNV ranging from −16° to 50°. The orientation of the LT showed to imply a weak influence in the width of the IFS. A total of 50 patients in this study met the radiographic criteria for IFI, the patients were asymptomatic for IFI. Three subjects demonstrated a positive LTV (3°, 9° and 17°) indicating an anterior oriented axis. The IFS in these three patients were within the normal range for asymptomatic patients demonstrated in previous literature [10, 12] and may be considered to have less probability of developing IFI. Similarly, patients that were found to have highly retroverted LTV also exhibited IFS within the asymptomatic range.

The high variation of the LTV observed in our study differs from the findings of Unlu *et al*. [1]. Those authors reported the LT to be retroverted on average −34.1° ± 3.0°, with 100% of the LTV differing from the mean by <5°. The present study also differs from the Unlu in regard to study cohort and methodology. Unlu determine the LTV in a group of 59 osteoarthritic hips utilizing a method that placed one centroid at the body of the LT to determine the LT axis. The present study utilized 250 asymptomatic hips. Two centroids were placed at the LT to determine the axis, which was proven to be statistical consistent among the measurements. Similar to Unlu *et al*. [1], the present investigation did not detect a gender difference in LTV (−23.6°, males and −24.5°, females).

The average angle formed between FNV and LTV was 38.4° ± 9.6° and demonstrated a moderate variability (30%) and correlation (0.63, *P* < 0.01). The relationship between the LT and FN axes found in our study was also different from the reported by Unlu *et al* [1]. Therefore, our findings recommend cautious utilization of the LT as a guide for insertion of femoral components in reconstructions surgeries.

FNV has been frequently associated to development of intra- and extra-articular hip pathologies [15, 22–29]. Siebenrock *et al*. [29] showed a high prevalence of posterior extra-articular impingement at the LT and at the ischial tuberosity in subjects with presence of hip valgus and increased FNV [29]. These findings support two separate studies concerning the relationship and implications of FNV in developing IFI [13, 14]. Bredella *et al*. [13] performed a MRI investigation of morphologic factors that may predispose IFI and found that symptomatic subjects with IFI presented with an increased FNV compared with control subjects (19.7° ± 11.1° versus 15.5° ± 12.1°). Another study by Gomez-Hoyos *et al.* found the presence of increased FNV (21.7° versus. 14.1°) and LTFN (45.4° versus 38.3°) in symptomatic subjects for IFI compared with asymptomatic subjects [14]. There were no differences between studies when comparing the LTV. This finding helps to understand that FN and LT share the same rotational axis, with FNV acting as a key morphologic factor contributing to development of IFI. The comprehension of IFI as a result of the interaction of three-planar axis is necessary for diagnosis and treatment decisions.

The relationship between FNV and LTV could have potential clinical relevance to the iliopsoas musculotendinous unit. Fabricant *et al*. [15] showed that increased femoral version may influence the iliopsoas kinematics and clinical outcomes after arthroscopic lengthening of symptomatic snapping hip. The authors did not consider the LT anatomy as an element that may influence outcomes. Recently, Gomez-Hoyos *et al*. [6] showed that subjects diagnosed with snapping hip presented with increased restroversion of the LT compared with asymptomatic subjects (−31.1° versus −24.2°). The LT is the insertion site for the iliopsoas muscle and the relationship between LTV and FNV could affect hip kinematics and stabilization.

Among the 250 asymptomatic hips in this study, the average IFS measured 22.9 ± 7.0 mm. The orientation of the LT showed a weak influence on the width of the IFS (*r* = −0.16, *P* < 0.05). Johnson was the first author to describe the IFS as the distance between the LT and the ischium when the hip is in slight adduction, external rotation, and extension [30]. More recently, Torriani *et al*. [10] quantified the IFS in a MRI study of patients diagnosed with IFI. The presence of IFS with a cut-off value <17 mm could represent a potential risk to impingement of the LT against the ischial tuberosity. Several studies have utilized this cut-off value as an imaging criteria for the diagnosis of IFI [5, 7, 8, 12]. This value still controversial and not totally clear due to the anatomic and dynamic conditions involving IFI [31, 32].

The present study demonstrated that the width of IFS is not related with the axial orientation of the LT. Some predisposing factors that may contribute to decreased IFS have been cited in the literature, such as: coxa valga, osteochondromas of the pelvis, intertrochanteric fractures, alterations of biomechanics due to total hip arthroplasty [3, 7, 10, 11]. An altered osseous anatomy with increased FNV and ischial angle are factors recently found to decrease IFS in symptomatic subjects with IFI [13, 14]. Similarly, morphologic differences between genders can be attributed to influence in decreasing the IFS. Female subjects tend have a wider pelvis, a lower neck-shaft angle and more FN inclination [12, 33]. These characteristics may help understand the high prevalence of IFI in female population among the studies [7, 8, 10, 12, 13, 31].

The findings of the present study highlight the necessity to understand the IFS in three planar axis and the kinematic chain. Finnoff *et al*. [11] demonstrated a statistically significant difference in IFS according to hip position. A largest IFS occurred with the hip in abduction and internal rotation, whereas hip adduction and external rotation resulted in the narrowest IFS. Gait abnormalities with pelvic drop and excessive adduction of the femur during the stance phase can result from hip intra extra-articular pathology, abductor muscle impairment and weakness [34, 35]. These factors may represent a risk for decreasing the IFS, and consequently impinging the LT against the ischium during terminal extension with dynamic load [7, 14, 29]. The long stride-walking test and the IFI test utilize the same dynamic concept to recreate the impingement and diagnose IFI with high specificity and reproducibility. Both tests are performed in order to recreate the dynamic impingement in an active and passive motion that combines hyperextension, adduction and external rotation of the femur [36]. The utilization of the IFI and long stride walking tests for the prompt diagnosis of IFI are supported in the study performed by Sussman *et al*. [37]. Radiological changes at the quadratus femoris muscle were not related to the width of the IFS, but with the proximity of the insertion site of the quadratus femoris muscle and intratuberous distance which could influence muscle function.

The limitations of this study include its retrospective nature and patient selection. While measurements were taken on asymptomatic hips, the patients did have symptoms involving the contralateral non-measured side. Future studies should consider measurements in subjects clinically normal to determine the relationship between these findings with normal values as well as symptomatic population to determine the lesser trochanteric orientation and its relationship with hip pathologies previous discussed in this study. Within this sample of 250 subjects, females composed the majority of the subjects, due to the morphological differences between sexes that were previously mentioned, analysis of more MRI from male subjects are necessary.

In conclusion, among 250 asymptomatic hips the LTV showed a high variation with a moderate relationship with the FNV. The LTV do not influenced the width of the IFS. The measurements of LTV, FNV and IFS may offer an additional set of data for diagnosis and treatment considerations. Ischiofemoral impingement is likely a multidimensional problem, which involves consideration of the entire femur and should be assessed on a three-planar biomechanical axis. An understanding of LTV and its dynamic implications in combination with physical examination testing could provide additional insight on the biomechanics of IFI.
